# Integrated bioinformatics analysis and experimental validation reveal Pevonedistat as a promising therapeutic agent modulating the CRL4–DTL–p21/p53 axis in nasopharyngeal carcinoma

**DOI:** 10.1186/s41065-026-00661-2

**Published:** 2026-03-07

**Authors:** Cong-Chao Jia, Zhen-Yi Li, Wei-Hua Jia, Cheng-Tao Jiang

**Affiliations:** 1 State Key Laboratory of Oncology in South China, Guangdong Key Laboratory of Nasopharyngeal Carcinoma Diagnosis and Therapy, Guangdong Provincial Clinical Research Center for Cancer, Sun Yat-sen University Cancer Center, Guangzhou, 510060 P.R. China; 2 Clinical Medical College, Southwest Medical University, Luzhou, 646000 P. R. China; 3 School of Public Health, Sun Yat-sen University, Guangzhou, 510080 P.R. China; 4 Department of Anesthesiology, State Key Laboratory of Oncology in South China, Collaborative Innovation for Cancer Medicine, Sun Yat-sen University Cancer Center, Guangzhou, Guangdong 510060 P. R. China

**Keywords:** Nasopharyngeal carcinoma, DTL, Pevonedistat, Machine learning, Molecular docking, PDX models

## Abstract

**Background:**

Nasopharyngeal carcinoma (NPC) continues to represent a significant therapeutic challenge. Despite the substantial success of radiotherapy, chemotherapy, and immunotherapy, their effectiveness remains limited in recurrent or advanced NPC. Hence, the urgent identification of new molecular targets and effective therapeutic agents is critically required.

**Methods:**

Bulk and single-cell transcriptomic data were analyzed using bioinformatics approaches, and machine learning algorithms were employed to screen for hub genes in NPC. Functional validation of hub genes was performed through loss-of-function assays. Potential therapeutic agents were identified through molecular docking and subsequently evaluated for anti-NPC efficacy using both in vitro NPC cell line models and in vivo patient-derived xenograft (PDX) models.

**Results:**

Bioinformatics analyses revealed *DTL* as a central gene with critical diagnostic and prognostic significance. Knockdown of *DTL* led to cell cycle arrest and apoptosis through stabilization of p21 and p53. Pevonedistat markedly inhibited DTL activity, recapitulating the effects of *DTL* knockdown. In vitro studies showed that Pevonedistat suppressed NPC cell proliferation, while in PDX models it significantly reduced tumor burden. Collectively, the data establish *DTL* as a key oncogenic driver in NPC and highlight Pevonedistat as a promising therapeutic candidate.

**Conclusion:**

Our work presents an integrated framework for target identification and therapeutic development in NPC. These findings deepen our understanding of NPC biology and highlight that Pevonedistat suppresses cell proliferation and tumor growth of NPC via the CRL4-DTL-p21/p53 axis.

**Graphical Abstract:**

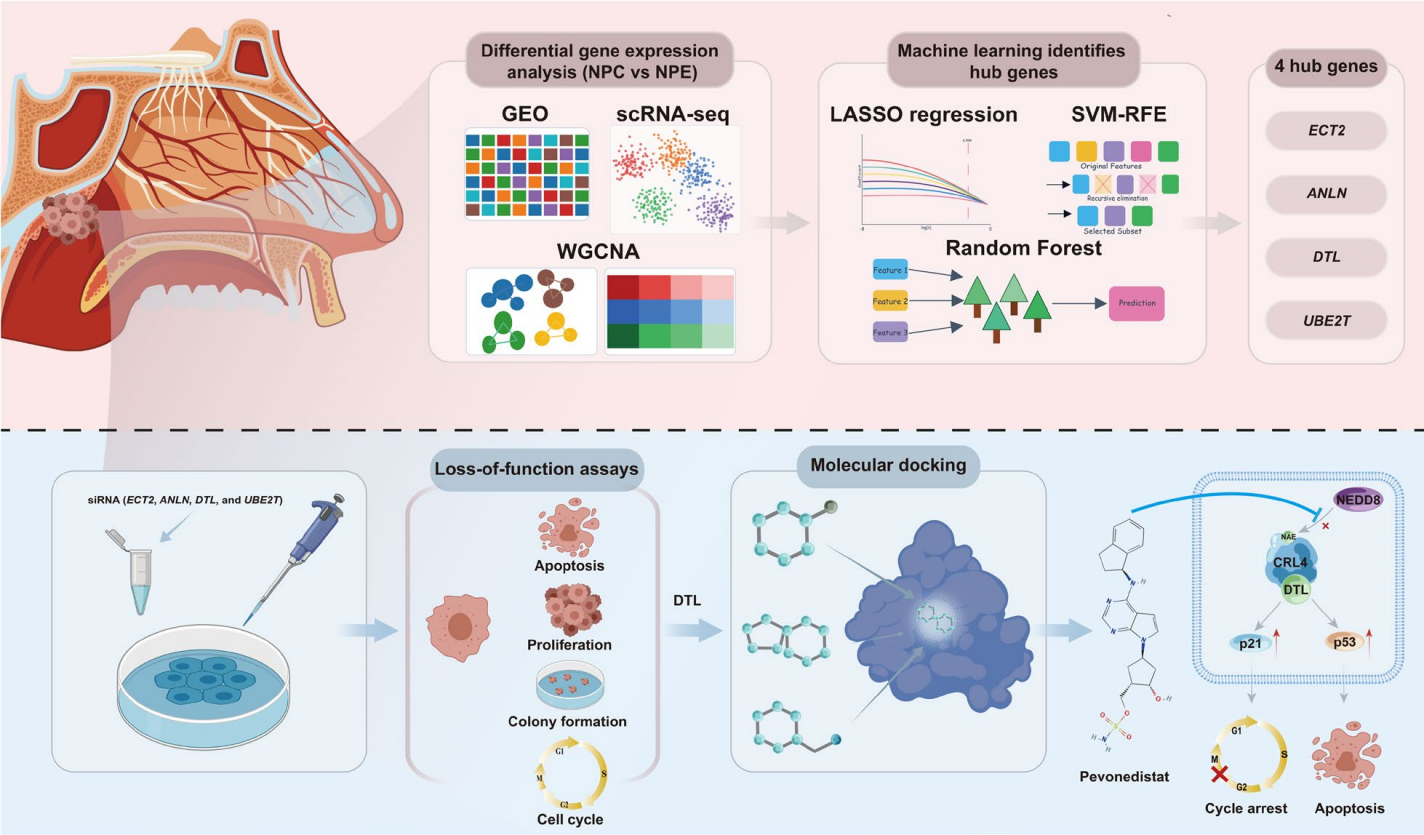

**Supplementary Information:**

The online version contains supplementary material available at 10.1186/s41065-026-00661-2.

## Introduction

Nasopharyngeal carcinoma (NPC) is an epithelial malignancy that originates from the nasopharyngeal mucosa and most commonly arises in the pharyngeal recess [1, 2]. Despite sharing a similar histological origin, NPC differs from other epithelial head and neck cancers, being characterized by a non-keratinizing undifferentiated histological subtype, an endemic distribution, a strong association with Epstein–Barr virus (EBV), and marked aggressiveness [3–6]. Genetic predisposition, environmental exposures, lifestyle factors, and EBV infection are recognized as the major etiological contributors to NPC [7–9]. This malignancy exhibits a distinct geographic distribution, with particularly high prevalence in Southeast Asia and especially in the Guangdong and Guangxi provinces of Southern China [6]. Because of its anatomically concealed primary site and early tendency for lymphatic dissemination, 70% of patients are diagnosed at advanced stages when therapeutic options are limited [10, 11]. Although advances in radiotherapy, chemotherapy, and the rapid development of immunotherapy have improved local control rates, the long-term survival of patients with advanced NPC remains unsatisfactory [12–14]. Frequent recurrence, distant metastasis, and resistance to standard therapies underscore the urgent need to identify novel biomarkers and develop more effective therapeutic strategies to improve patient outcomes [14, 15]. Beyond the well-established genetic and molecular underpinnings of NPC, recent conceptual advances have increasingly emphasized the complexity of NPC as an ecological and evolutionary disease. In this framework, NPC is not viewed solely as a genetic disorder but as a dynamic pathological ecosystem, where tumor cells, the microenvironment, Epstein–Barr virus infection, and evolutionary selective pressures interact spatiotemporally to shape disease progression and therapeutic response. Such an ecological perspective integrates tumor heterogeneity, microenvironment interactions, and adaptive processes, offering a broader conceptual foundation for understanding NPC biology and the challenges associated with treatment [16].

A growing body of evidence suggests that dysregulation of key genes and signaling pathways plays a pivotal role in the initiation, progression, and therapeutic resistance of NPC [3, 17, 18]. Disrupted cell-cycle control, impaired DNA damage responses, epithelial–mesenchymal transition, and immune evasion have all been implicated in NPC pathogenesis [3, 19–22]. However, the molecular mechanisms driving these processes remain incompletely understood, and clinically actionable therapeutic targets are still lacking. With the rapid development of high-throughput sequencing technologies, multi-omic approaches such as transcriptomics and single-cell RNA sequencing (scRNA-seq) have provided valuable insights into the molecular heterogeneity of NPC [23–25]. Moreover, machine learning algorithms, including LASSO, Random Forest (RF), and Support Vector Machine-Recursive Feature Elimination (SVM-RFE), have facilitated the identification of potential targets from large-scale sequencing datasets [26, 27]. Despite these advances, most bioinformatics-predicted biomarkers have not undergone systematic functional validation, and only a limited number have progressed toward clinical application [28]. Therefore, comprehensive screening of functional genes and clarification of their biological roles are critical for the development of reliable diagnostic biomarkers and effective targeted therapies for NPC.

In this study, we combined bioinformatics and machine learning approaches to identify hub genes of NPC, followed by experimental validation that established DTL as a critical regulator. Molecular docking analysis further characterized Pevonedistat as a potential inhibitor of the CRL4-DTL complex. The antitumor efficacy of Pevonedistat was subsequently confirmed in both in vitro and in vivo models, alongside preclinical evaluation of its safety profile. Collectively, these findings establish DTL as an important therapeutic target in NPC and highlight Pevonedistat as a promising candidate for future clinical translation.

## Materials and methods

### Differential expression genes screening

To identify differential expression genes (DEGs) between NPC and normal nasopharyngeal epithelium (NPE), we analyzed the GSE53819 dataset using the “limma” package in R (version 4.3.1). DEGs were defined with thresholds of |log2FC| > 1 and adjusted P-value < 0.05. The “ggplot2” and “pheatmap” packages in R were applied to generate volcano plots and heatmaps, respectively, for the visualization of DEGs.

### Weighted gene co-expression network analysis (WGCNA)

Based on the GSE53819 dataset, group information was used as the trait, and WGCNA was performed using the “WGCNA” package in R. The top 5,000 genes with the highest variance were selected for analysis. To evaluate the overall correlation of samples in the training set, hierarchical clustering was conducted using the hclust function. The soft-thresholding power was determined with the pickSoftThreshold function, and hierarchical clustering based on the topological overlap matrix (TOM) was performed using the hierarchicalCluster function.

### Single cell RNA sequencing (scRNA-seq) analysis

Based on the nasopharyngeal carcinoma scRNA-seq dataset GSE150430, malignant and non-malignant cells were distinguished according to the previously published report [23]. Differentially expressed genes were identified between the two groups using thresholds of |log2FC| > 1 and adjusted *P* < 0.05.

### Enrichment analysis

To explore the biological functions and processes associated with the candidate genes, Gene Ontology (GO) and Kyoto Encyclopedia of Genes and Genomes (KEGG) enrichment analyses were performed using the “clusterProfiler” package in R.

### Protein-protein interaction (PPI) network analysis

To explore the protein-level interactions of the candidate genes, we uploaded the identified genes into the STRING database (https://cn.string-db.org), specified the species as “Homo sapiens” and conducted protein–protein interaction (PPI) network analysis in Cytoscape 3.9.1, applying an interaction score threshold of > 0.7. Furthermore, we used the cytohubba plugin in Cytoscape to assess node importance through ranking algorithms and selected the overlapping genes that ranked within the top 30 by maximal clique centrality (MCC), maximum neighborhood component (MNC), and degree as key candidate genes.

### Machine learning for hub genes

LASSO: LASSO regression analysis was performed on the sequencing training set using the “glmnet” package in R. A LASSO classification model was built based on binomial variables, using the lambda value corresponding to one standard error (1-SE criterion) to avoid overfitting. This approach aimed to prevent overfitting and improve predictive accuracy. Ten-fold cross-validation was used to identify feature genes associated with NPC diagnosis. Random forest (RF): Based on the expression profiles of candidate genes in the sequencing training set, the RF algorithm was implemented using the “randomForest” package in R. The top 10 candidate genes were selected as feature genes. Support vector machine-recursive feature elimination (SVM-RFE): In the sequencing training set, candidate genes were used to classify selected module genes with the SVM classifier in R. The SVM-RFE algorithm was implemented using the “caret” package in R. The number of folds for cross-validation was set to k = 10.

### Validation of hub gene expression

In the GSE53819 and GSE12452 datasets, expression differences of the core genes were evaluated between NPC and NPE samples. A t-test was applied to compare gene expression levels between NPC and control samples in both the training and validation sets, and the results were visualized using boxplots.

### Receiver operating characteristic (ROC) curve

The “pROC” package in R was employed to construct ROC curves using data from the GSE53819 and GSE12452 datasets. The diagnostic value of the core genes in NPC was assessed by calculating the area under the ROC curve (AUC).

### Cell lines and cell culture

The TW03 and HK1-EBV cell line was kindly provided by Prof. Musheng Zeng (Sun Yat-sen University Cancer Center), and the NPC43 cell line was generously provided by Prof. Lin Feng (Sun Yat-sen University Cancer Center). The NP69 cell line was purchased from SUNNCELL and maintained in a specialized medium according to the manufacturer’s instructions. TW03, NPC43, and HK1-EBV cells were cultured in RPMI-1640 medium (GIBCO, C11875500BT) supplemented with 10% fetal bovine serum (FBS, Biochannel), 100 U/mL penicillin, and 100 µg/mL streptomycin (Gibco). In addition, NPC43 cells were supplemented with 4 µM Y27632 (Selleck, 146986-50-7), and HK1-EBV cells were cultured with additional 700 µg/mL G418 (AmBeed, A261695). All cells were maintained at 37 °C in a humidified incubator with 5% CO₂. Cell line authentication and mycoplasma testing were performed to ensure experimental reliability.

### Quantitative real-time PCR

Total RNA was extracted using the EZ-press RNA Purification Kit (EZBioscience, B0004D). Reverse transcription was performed using 4× EZscript Reverse Transcription Mix II (EZBioscience, EZB-RT2GQ) according to the manufacturer’s instructions. Real-time PCR was conducted with SYBR Green qPCR Master Mix (EZBioscience, A0012-R2), and the primer sequences are provided in Supplementary Table 1. Gene expression was quantified using the ΔΔCt method, with GAPDH as the internal control. Data are presented as fold changes (2 − ΔΔCt). Statistical analysis was performed using a t-test, with *P* < 0.05 considered statistically significant.

### siRNA transfection

siRNA transfection was performed using Lipofectamine™ RNAiMAX Transfection Reagent (Invitrogen, 13778030) according to the manufacturer’s protocol. In brief, siRNA was mixed with the transfection reagent in Opti-MEM medium and incubated for 5 min at room temperature. The mixture was then added to cells cultured in RPMI-1640 medium. After 24–72 h, the cells were harvested for further analysis.

### CCK-8 Assay

Cell viability was assessed using the Cell Counting Kit-8 (APExBIO, K1018) following the manufacturer’s instructions. Briefly, 2 × 10^3^ cells were seeded in 96-well plates and cultured for 8 h to allow cell attachment. The cells were then treated with drug or siRNA at various concentrations for 72 h. After treatment, 10 µL of CCK-8 solution was added to each well, and the cells were incubated at 37 °C for 2 h. The absorbance at 450 nm was measured using a microplate reader. Cell viability was calculated as a percentage relative to the control group.

### Flow cytometry analysis of cell cycle

Cell cycle distribution was analyzed by flow cytometry following propidium iodide (PI) staining. Briefly, cells were seeded in culture dishes and treated with siRNA or drug at various concentrations for 24–72 h. After treatment, cells were fixed with 70% ethanol at -4 °C for at least 24 h. Following fixation, cells were washed with PBS and incubated with PI and RNase A (LEAGENA, DA0030) in PBS for 30 min at 37 °C in the dark. The cell cycle distribution was analyzed using a CytoFLEX flow cytometer (Beckman Coulter), and the percentages of cells in the G0/G1, S, and G2/M phases were determined.

### Flow cytometry analysis of cell apoptosis

Apoptosis was assessed using the eBioscience™ Annexin V Apoptosis Detection Kit (Thermo Fisher Scientific, 88-8005-74) in accordance with the manufacturer’s instructions. Briefly, cells treated with either the drug or siRNA were collected, washed twice with cold PBS, and resuspended in binding buffer. Annexin V-FITC and PI were subsequently added, and the samples were incubated for 15 min at room temperature in the dark, followed by immediate analysis using a CytoFLEX flow cytometer (Beckman Coulter). The proportions of viable, early apoptotic, late apoptotic, and necrotic cells were quantified.

### Colony formation assay

Cells were seeded in 6-well plates at a density of 1,000 cells per well and cultured in complete medium at 37 °C in a humidified incubator with 5% CO₂. After 12 h of attachment, the cells were treated with either the drug or siRNA and cultured for 5–10 days until visible colonies had formed. Colonies were subsequently fixed with 4% paraformaldehyde for 15 min, stained with 0.1% crystal violet for 20 min, and rinsed with PBS. After air-drying, colonies containing more than 50 cells were counted under a microscope. The colony formation rate was calculated relative to that of the control group.

### Molecular docking

The three-dimensional structures of compounds were retrieved from the PubChem database, and protein crystal structures were obtained from the RCSB Protein Data Bank. Molecular docking analyses were conducted using the CB-Dock2 platform [29]. Binding energies were calculated for each predicted conformation, with more negative values reflecting stronger binding affinity. In general, binding energies lower than − 5.0 kcal/mol were considered indicative of stable molecular interactions. Final docking conformations were subsequently visualized and further analyzed using PyMOL and Discovery Studio to evaluate potential hydrogen bonding and hydrophobic interactions of the ligands within the binding pocket.

### Western blot

Total protein was extracted with RIPA lysis buffer (Beyotime, P0013B) containing protease and phosphatase inhibitors. Protein concentration was measured using the BCA Protein Assay Kit (Thermo Fisher Scientific, 23227). Equal protein amounts were resolved by SDS–PAGE and transferred to PVDF membranes (Millipore, IPVH00010). Membranes were blocked in 5% non-fat milk/TBST for 1 h at room temperature and incubated overnight at 4 °C with primary antibodies. After washing, membranes were incubated with HRP-conjugated secondary antibodies for 1 h at room temperature. Details of the antibodies are listed in Supplementary Table 2. Protein bands were visualized using an enhanced chemiluminescence (ECL) kit (Thermo Fisher Scientific, 32106).

### Establishment and transplantation of C17 PDX

The patient-derived nasopharyngeal carcinoma xenograft C17 (C17-PDX) was generously provided by Prof. Pierre Busson (Institut Gustave Roussy, France) and subsequently obtained through Prof. Musheng Zeng (Sun Yat-sen University Cancer Center). The C17 tumor has been maintained in nude mice through continuous serial subcutaneous transplantation. All experiments in this study were performed with the 31st generation of the C17-PDX. For transplantation, mice were euthanized humanely when tumors reached the plateau phase, defined by stabilized growth or surface ulceration. Tumors were excised carefully, with the capsule and necrotic central regions removed to preserve viable compact tissue. The excised tumor tissue was cut into fragments of approximately 1–2 mm³, washed three times with PBS, and rinsed an additional three times in RPMI-1640 medium supplemented with antibiotics. The resulting fragments were subsequently transplanted into recipient mice for further propagation.

### Animal experiments

All animal experiments were conducted in accordance with the institutional guidelines and approved by the Animal Ethics Committee of Sun Yat-sen University Cancer Center (Approval No. 2022 − 000163). Male BALB/c nude mice (6–8 weeks old) were obtained from GemPharmatech (Guangzhou, China) and housed under specific pathogen-free (SPF) conditions. After one week of acclimatization, mice were randomly assigned to control and treatment groups, and the C17-PDX model was established as described. From day 6 post-transplantation, Pevonedistat (10 mg/kg) or vehicle was administered via peritumoral injection every three days. Tumor volume was monitored every three days using calipers and calculated as V = (length × width²)/2. At the endpoint, mice were euthanized, tumors were excised for imaging and weighing, and blood and major organs were collected for hematological, biochemical, and histopathological analyses to evaluate toxicity.

## Results

### Integrated bioinformatics analyses to identify genes associated with NPC.

**Fig. 1:**
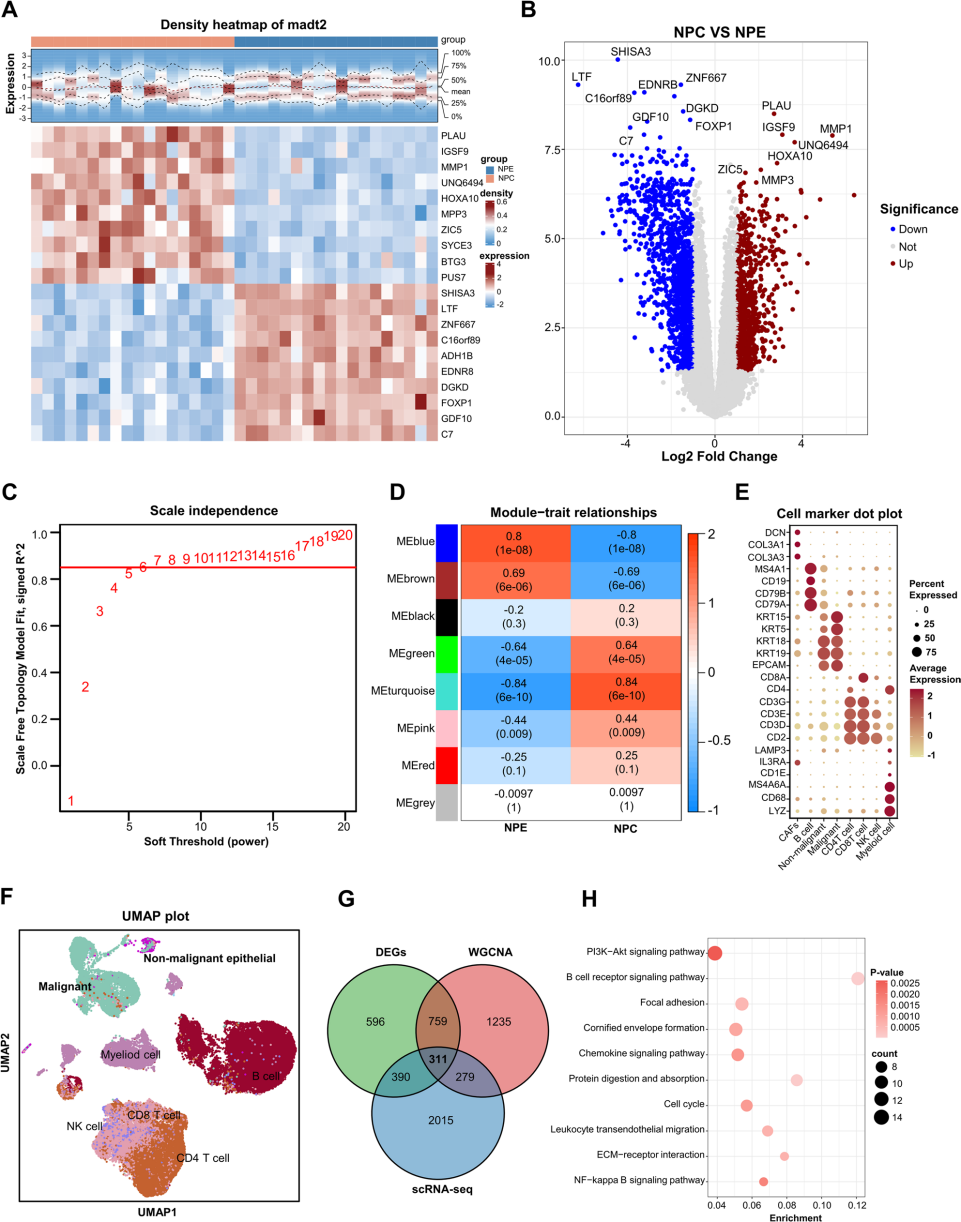
Integrated bioinformatics analyses to identify genes associated with nasopharyngeal carcinoma (NPC). **A** Differentially expressed genes (DEGs) between nasopharyngeal carcinoma (NPC, *n* = 18) and nasopharyngeal epithelial tissues (NPE, *n* = 18) in the GSE53819 dataset. **B** Volcano plot and heatmap highlighting significantly upregulated and downregulated genes, with thresholds set at |log2 fold change| > 1 and adjusted *P* < 0.05. **C** Scale-free topology fit index and mean connectivity analysis across a range of soft-thresholding powers in weighted gene co-expression network analysis (WGCNA). **D** Heatmap of module–trait relationships showing correlations between gene modules and NPC status. Correlation coefficients and corresponding P values are shown in each cell. **E** Dot plot showing the expression of canonical cell-type markers in single-cell RNA sequencing (scRNA-seq) data from NPC samples. Average expression levels and the proportions of marker-positive cells are displayed for epithelial, T, B, NK, and myeloid cell populations. **F** Uniform manifold approximation and projection (UMAP) of scRNA-seq data, illustrating the distribution of malignant epithelial cells, non-malignant epithelial cells, CD4⁺ and CD8⁺ T cells, B cells, NK cells, and myeloid cells. **G** Venn diagram illustrating the overlap among DEGs, hub genes identified by WGCNA modules, and differentially expressed genes derived from scRNA-seq. **H** KEGG pathway enrichment analysis of the overlapping genes. Significantly enriched pathways were determined using a hypergeometric test with false discovery rate (FDR) correction (*P* < 0.05)

To identify DEGs between NPC and NPE, differential expression analysis was performed on the dataset GSE53819 using the “limma” package in R. DEGs were defined with thresholds of |log2FC| > 1 and adjusted P value < 0.05. Volcano and heatmaps were generated to visualize the DEGs, with the most significantly altered genes clearly labeled. As shown in Fig. 1A-B, a total of 2,056 DEGs were identified, including 784 upregulated genes and 1,272 downregulated genes in NPC samples. To identify gene modules associated with NPC, WGCNA was performed, with group information set as the trait in the training dataset. The top 5,000 genes with the highest variance were selected for analysis. To evaluate the overall sample correlation, hierarchical clustering was conducted using the hclust function, and two outlier samples were removed (Supplementary Fig. 1). To determine an appropriate soft-thresholding power, the pickSoftThreshold function was applied. A soft-thresholding power of 6 was selected to approximate a scale-free topology with R² = 0.85 (Fig. 1C). The adjacency matrix was subsequently transformed into a topological overlap matrix (TOM) to reduce noise and spurious correlations. Hierarchical clustering of the TOM-based dissimilarity matrix was performed using the hierarchicalCluster function, with the minimum module size set to 100 genes. A total of nine co-expression gene modules were identified, as shown in the clustering dendrogram (Fig. 1D). Of the identified modules, the green and turquoise modules showed the strongest associations with NPC. These were selected for further investigation, encompassing 2,584 genes in total. We next analyzed publicly available scRNA-seq dataset (GSE150430) from NPC samples. Clustering was performed based on the markers shown in Fig. 1E, and the resulting cell populations are illustrated in Fig. 1F. Our analysis primarily focused on differences between malignant and non-malignant cells, which led to the identification of 2,995 differentially expressed genes. As illustrated in Fig. 1G, the intersection of DEGs, genes from the two WGCNA modules, and the differentially expressed genes identified between malignant and non-malignant epithelial cells from scRNA-seq analysis was subsequently obtained, yielding a total of 311 genes. Functional enrichment analysis of the 311 genes was conducted, with KEGG pathway results displayed in Fig. 1H and GO enrichment outcomes provided in Supplementary Fig. 2. Enriched pathways included the PI3K–AKT signaling cascade, cell cycle regulation, and other related biological processes.

### Screening and validation of hub genes in NPC based on machine learning

**Fig. 2:**
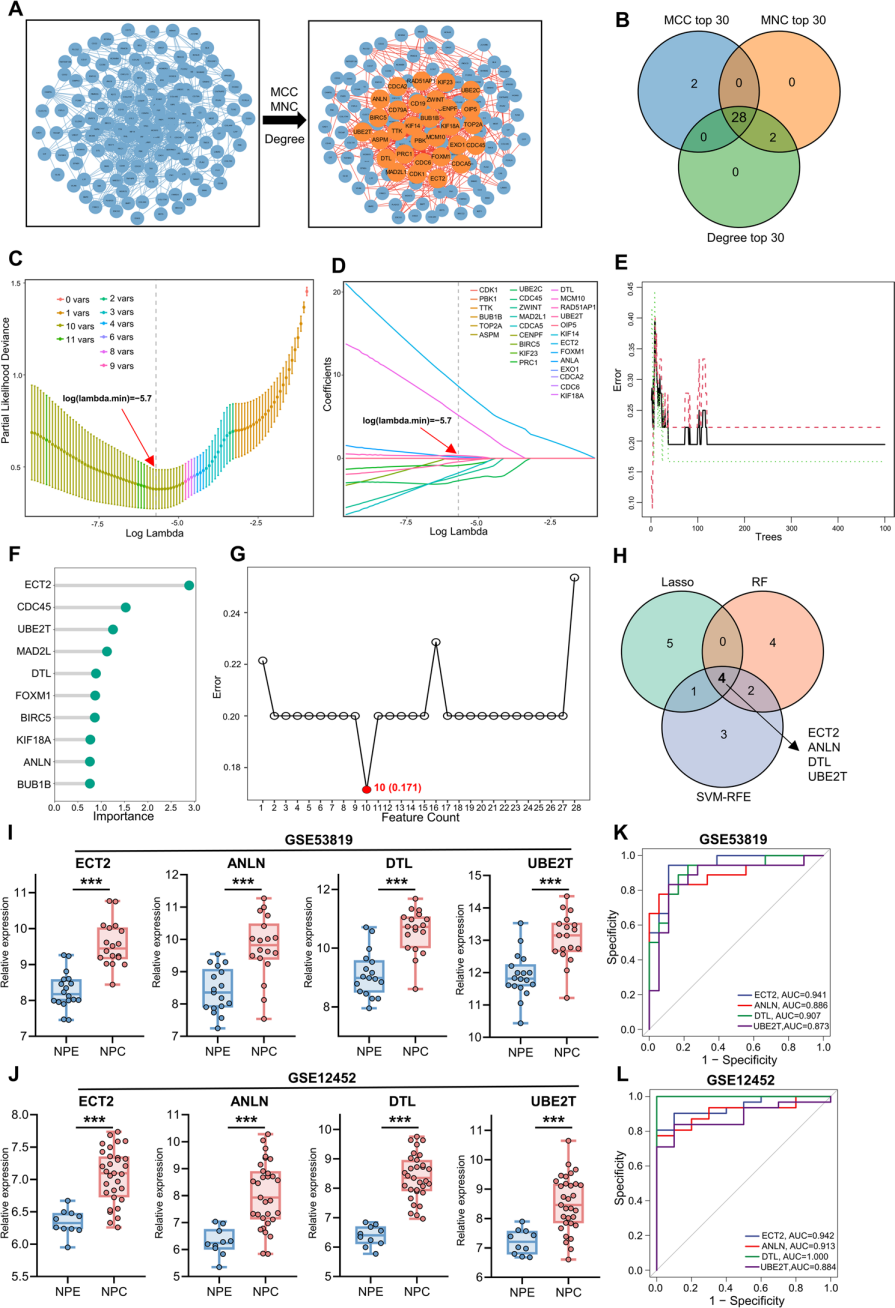
Screening and validation of hub genes in NPC based on machine learning. **A** Topological analysis of the PPI network to determine hub genes in NPC. MCC: maximal clique centrality. MNC: maximum neighborhood component. **B** Intersection of the top 30 genes ranked by MCC, MNC, and degree values. **C** Cross-validation error curve of the LASSO regression model. The x-axis represents the logarithm of the penalty parameter λ, and the y-axis denotes the partial likelihood deviance. The optimal λ (lambda.min) was determined at log(λ) = –5.7, where the cross-validation error reached its minimum. **D** Coefficient path plot of the LASSO regression model. The x-axis represents the logarithm of the penalty parameter λ, and the y-axis indicates the regression coefficients of individual genes. Each colored line corresponds to a gene. As λ increases, most gene coefficients shrink toward zero. **E** Relationship between the number of decision trees and cross-validation error in the random forest (RF) model. The x-axis represents the number of trees in the random forest, and the y-axis denotes the cross-validation error rate. **F** The x-axis represents gene importance scores, and the y-axis denotes gene names. The top 10 most important genes are shown. **G** Cross-validation error curve of the random forest model. The x-axis represents the number of selected features (variables), and the y-axis denotes the cross-validation error rate. As the number of features increases, the error rate gradually decreases, reaching its lowest value (0.17) at 10 features, indicating that the model achieves optimal predictive performance while maintaining a balance between complexity and accuracy. **H** Intersection of feature genes selected by LASSO, RF, and SVM-RFE methods. **I**–**J** Expression levels of ECT2, ANLN, DTL, and UBE2T in NPC and NPE samples. Data are presented as mean ± SD. *** P＜0.001, compared with the NPE group. (K–L) ROC curves of ECT2, ANLN, DTL, and UBE2T in the GSE53819 and GSE12452 datasets.

To further screen the hub genes of NPC, we performed a protein–protein interaction analysis on the 311 previously identified genes and constructed a PPI network (Fig. 2A). The network was subsequently subjected to topological analysis, in which the MCC, MNC, and degree values of each gene were calculated. We then selected the top 30 genes ranked by MCC, MNC, and degree values, and their intersection yielded 28 genes (Fig. 2B), which were used for subsequent machine learning-based screening. To further screen and validate the candidate genes, three machine learning models were applied, including LASSO, RF, and SVM-RFE. We first conducted LASSO regression cross-validation, which determined the optimal lambda value at the minimum cross-validation error, as illustrated in Fig. 2C (log(lambda.min) = − 5.7). At this threshold, a subset of genes, including *CDK1*, *UBE2C*, *DTL*, and *ECT2*, maintained non-zero coefficients, thereby being identified as key feature genes (Fig. 2D). RF can construct a classification model, and simultaneously evaluate and rank the importance of genes to the model, thereby identifying critical genes for classification in the form of importance scores. As illustrated in Fig. 2E, the classification error progressively declined and reached a stable range (0.15–0.25) as the number of decision trees increased, suggesting robust predictive stability of the random forest model. Using importance measures derived from the random forest, the most critical feature genes were subsequently identified (Fig. 2F). Support vector machine (SVM) is a widely used supervised machine learning technique for classification and regression, while the recursive feature elimination (RFE) algorithm can identify the optimal combination of variables to maximize model performance. Therefore, the present study applied the SVM-RFE algorithm to identify feature biomarkers with superior discriminatory power. As shown in Fig. 2G, the model error reached its minimum value (0.171) when 10 genes were retained, indicating that this subset of genes provided the optimal classification performance and could serve as key features for subsequent analyses. Subsequently, we intersected the genes identified by the three machine learning methods described above, including LASSO, RF, and SVM-RFE, and ultimately obtained four genes, namely *ECT2*, *ANLN*, *DTL*, and *UBE2T* (Fig. 2H). Finally, we validated the expression levels of *ECT2*, *ANLN*, *DTL*, and *UBE2T* in the GSE53819 and GSE12452 datasets. As shown in Fig. 2I–J, these four genes were highly expressed in NPC samples and exhibited significant differences. Likewise, ROC curve analyses and AUC calculations based on the GSE53819 and GSE12452 datasets demonstrated that *ECT2*, *ANLN*, *DTL*, and *UBE2T* exhibited strong diagnostic performance, supporting their potential as diagnostic biomarkers (Figs. 2–L). In conclusion, using machine learning approaches, we identified *ECT2*, *ANLN*, *DTL*, and *UBE2T* as hub genes in NPC.

### Knockdown of hub genes induced cell cycle arrest and apoptosis in NPC cells

**Fig. 3:**
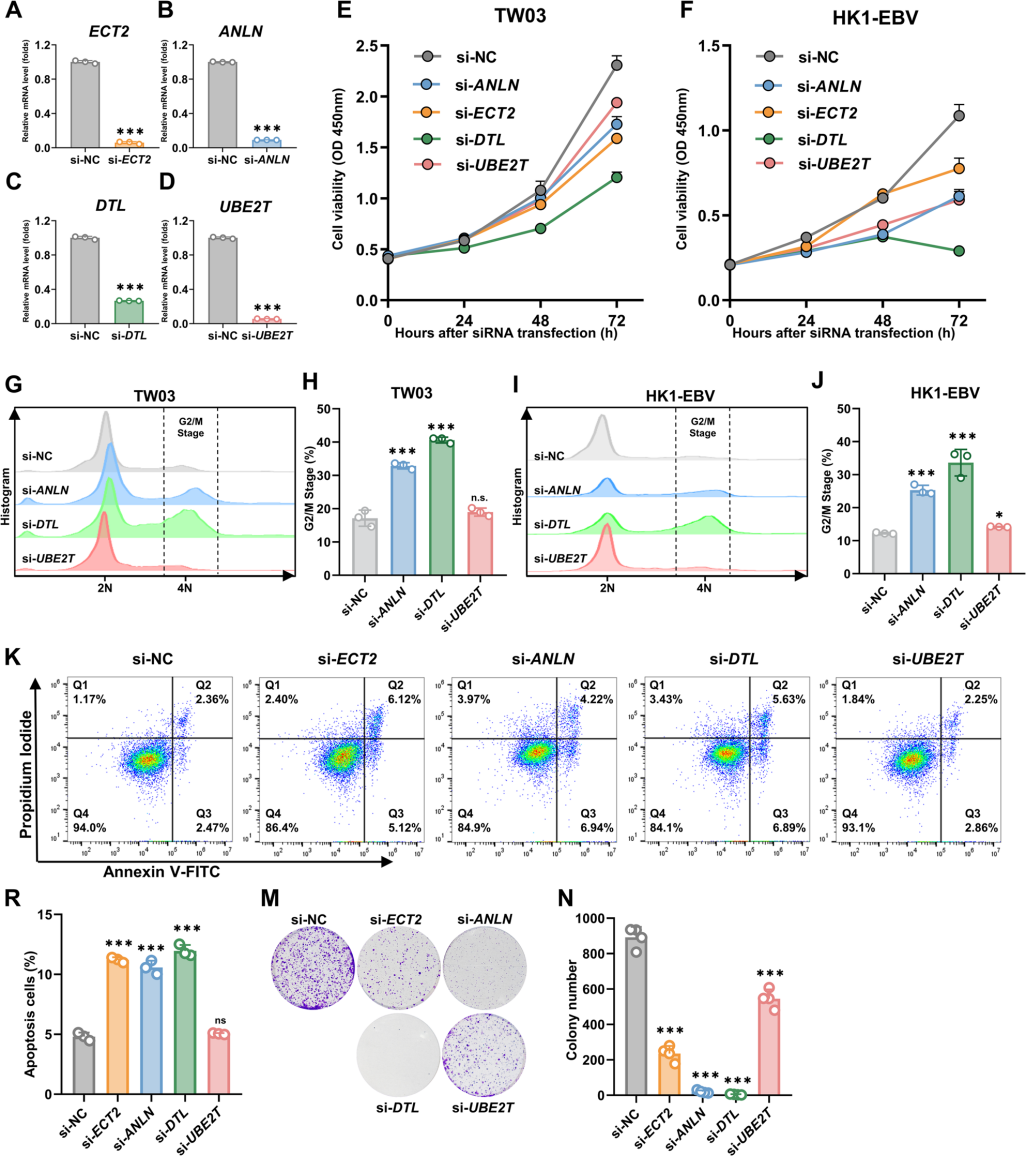
Knockdown of hub genes induced cell cycle arrest and apoptosis in NPC cells. **A**–**D** Validation of the knockdown efficiency of different siRNAs (*ECT2*, *ANLN*, *DTL*, and *UBE2T*). **E**–**F** TW03 and HK1-EBV nasopharyngeal carcinoma cell lines were transfected with different siRNAs, and cell viability was measured at different time points (0, 24, 48, 72 h) using the CCK-8 assay. **G**,** I** Representative flow cytometry histograms of cell cycle distribution in TW03 and HK1-EBV cells transfected with si-NC or siRNAs targeting ANLN, DTL, or UBE2T. **H**,** J** Quantification of the percentage of cells in the G2/M phase. **K–L** Flow cytometry analysis of the effect of different siRNA transfections on apoptosis, as determined by Annexin V-FITC and PI staining (*n* = 3). **M**–**N** Effect of different siRNA transfections on cell clonogenic ability (*n* = 4). All data are presented as mean ± SD from at least three independent experiments. Statistical analysis was performed using an unpaired two-tailed Student’s *t*-test. *** *P*<0.001, ** *P*<0.01, * *P*<0.05; n.s., not significant. All comparisons were made relative to the si-NC group

To investigate the functional roles of the four hub genes (*ECT2*, *ANLN*, *DTL*, and *UBE2T*) identified in the prior screening, we designed specific siRNAs to knock down their expression. The sequences of these siRNAs are shown in Supplementary Table 3. The knockdown efficiency of the siRNAs was validated in TW03 cells, as shown in Fig. 3A–D, where all four siRNAs markedly reduced the expression of their respective target genes. Cell proliferation was subsequently assessed by CCK-8 assays in two nasopharyngeal carcinoma cell lines, TW03 and HK1-EBV. As shown in Fig. 3E–F, silencing of each of the four genes suppressed cell proliferation, with the strongest inhibitory effect observed in the DTL-knockdown group. Previous studies have suggested that *ECT2*, *ANLN*, *DTL*, and *UBE2T* are functionally associated with cell cycle regulation [30–33]. Subsequently, we performed flow cytometry to assess the impact of their knockdown on the cell cycle distribution in nasopharyngeal carcinoma cells. As shown in Fig. 3G–J, knockdown of *ANLN* and *DTL* induced cell cycle arrest, as evidenced by an increased proportion of cells at the 4 N DNA content peak, whereas no marked changes were observed in the *UBE2T*-knockdown group (The original images are presented in Supplementary Fig. 3). Moreover, knockdown of *ECT2* markedly altered cell morphology, resulting in a distinct baseline compared with the other groups, which made reliable analysis difficult; therefore, the results for the *ECT2*-knockdown group are not shown. Given that prolonged cell-cycle arrest can drive apoptosis, we next asked whether silencing these genes sensitizes TW03 cells to undergo programmed cell death. As shown in Fig. 3K–L, silencing of *ECT2*, *ANLN*, and *DTL* led to different degrees of apoptosis induction, whereas the *UBE2T*-knockdown group showed no apparent increase in apoptotic cells. In addition, the morphology of TW03 cell at 36 h after transfection with different siRNAs is shown in Supplementary Fig. 4, where the *DTL*-silenced group exhibited the slowest growth and the poorest condition. We next assessed the effects of gene silencing on the clonogenic potential of TW03 cells. As shown in Fig. 3M–N, silencing of *ECT2*, *ANLN*, *DTL*, and *UBE2T* reduced colony formation to varying extents, with the strongest suppression observed in the *DTL*-knockdown group.

### Pevonedistat modulates the DTL–p21/p53 axis in NPC.

**Fig. 4:**
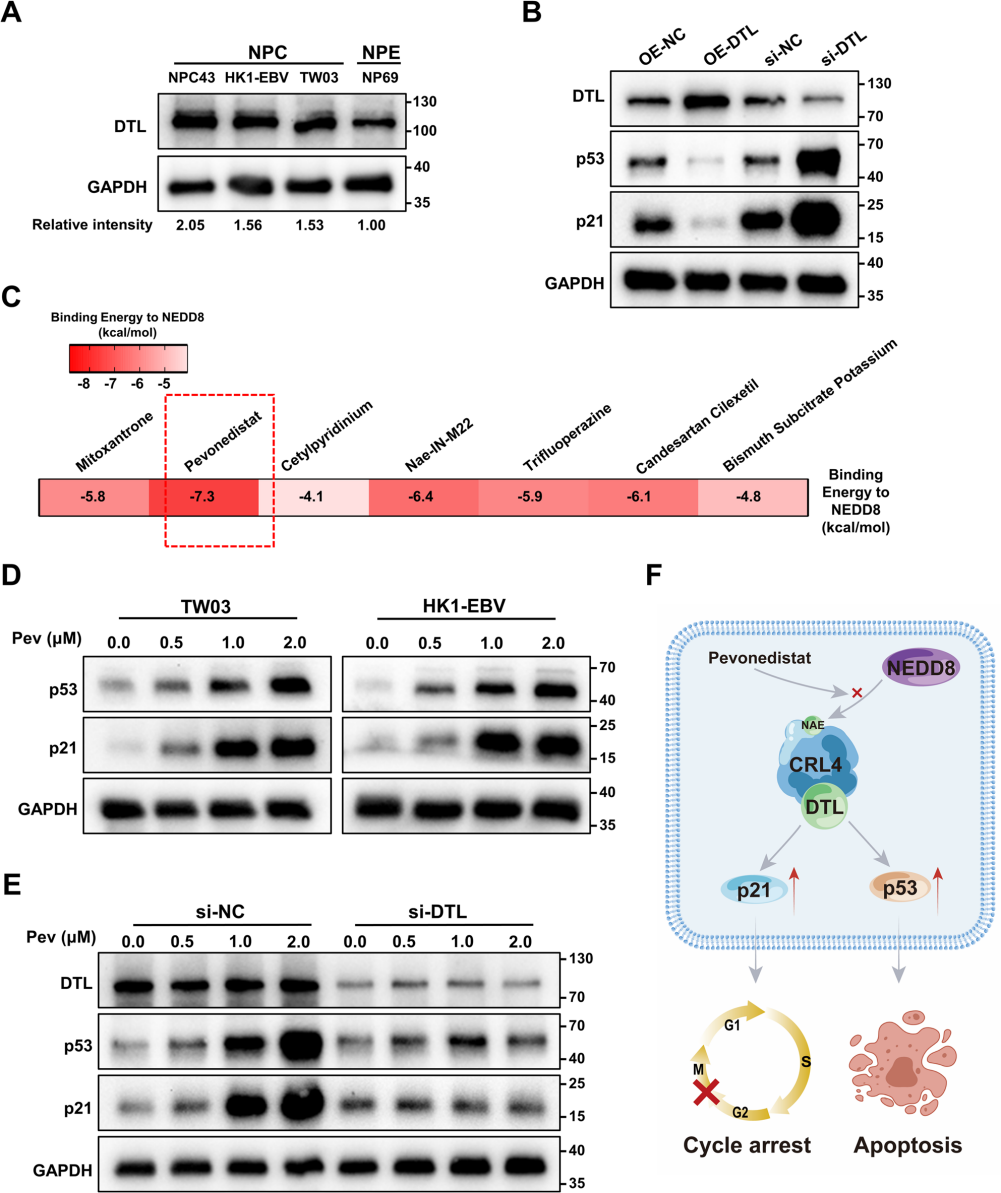
Pevonedistat modulates the DTL–p21/p53 axis in NPC. **A** Western blot analysis of DTL protein expression in nasopharyngeal carcinoma cell lines (NPC43, HK1-EBV, TW03) and normal nasopharyngeal epithelial cells (NP69), with GAPDH as an internal control. The relative expression levels were 2.05, 1.56, 1.53, and 1.00, respectively. **B** Western blot showing the effects of DTL overexpression and silencing on the protein levels of p21 and p53 in TW03 cells. **C** Comparison of predicted binding energies of candidate compounds docked to NEDD8. Binding energies (kcal/mol) were calculated by molecular docking, with lower values indicating stronger predicted binding affinity. **D** Pevonedistat induces p53 and p21 protein levels in nasopharyngeal carcinoma cells. **E** DTL knockdown attenuates Pevonedistat-induced p53 and p21 upregulation. Pev: Pevonedistat. **F** Schematic illustration of the proposed mechanism by which Pevonedistat regulates the CRL4-DTL-p21/p53 axis

We validated the expression of DTL protein in three NPC cell lines (TW03, HK1-EBV, and NPC43) and one NPE cell line (NP69) (Fig. 4A). Consistent with our earlier findings, DTL was overexpressed in NPC compared with NPE, highlighting its potential role in NPC pathogenesis. Functional assays in TW03 cells further revealed that DTL overexpression promoted the degradation of p21 and p53, whereas DTL knockdown increased their levels, suggesting that DTL may contribute to cell cycle dysregulation and uncontrolled proliferation by targeting these key regulators (Fig. 4B). Based on these results, we hypothesized that DTL represents a promising therapeutic target in NPC. To identify potential inhibitors, we retrieved candidate compounds from ChEMBL, DrugBank, and the Therapeutic Target Database [34–36]. While no direct CRL4–DTL inhibitors were retrieved, we identified seven small molecules that functionally inactivate CRL4–DTL by blocking upstream NEDD8-mediated neddylation. Molecular docking was performed to identify the most potent inhibitor among these seven compounds. Molecular docking analysis with NEDD8 (PDB ID: 4FBJ) indicated that Pevonedistat exhibited the strongest binding affinity, with a binding energy of − 7.3 kcal/mol (Fig. 4C). Detailed interaction analysis showed that Pevonedistat formed stable hydrogen bonds with Gln40, Arg42, Ala72, and Arg74 (Supplementary Fig. 5), implicating these residues in the binding process. Additional docking results of four other compounds with binding energies below − 5 kcal/mol are provided in Supplementary Fig. 6. Consistent with the docking predictions, Pevonedistat treatment led to a concentration-dependent increase in p21 and p53 protein levels in both TW03 and HK1-EBV cells (Fig. 4D). To further examine whether the effects of Pevonedistat on p21 and p53 are associated with DTL, TW03 cells were transfected with control siRNA (si-NC) or siRNA targeting DTL (si-DTL) prior to Pevonedistat treatment. As shown in Fig. 4E, DTL knockdown attenuated the Pevonedistat-induced upregulation of p21 and p53 compared with the si-NC group. Collectively, these findings suggest that DTL contributes, at least in part, to the regulation of the p53/p21 pathway upon Pevonedistat treatment. To further assess whether the antitumor effects of Pevonedistat are functionally associated with DTL, cell viability was evaluated using a CCK-8 assay. Pevonedistat-induced reduction in cell viability was markedly attenuated in DTL-silenced cells compared with the si-NC group (Supplementary Fig. 7). Taken together, these results support a model in which Pevonedistat modulates the p21/p53 pathway through a DTL-dependent mechanism, as schematically illustrated in Fig. 4F.

### In vitro validation of the anti-NPC effects of Pevonedistat

**Fig. 5:**
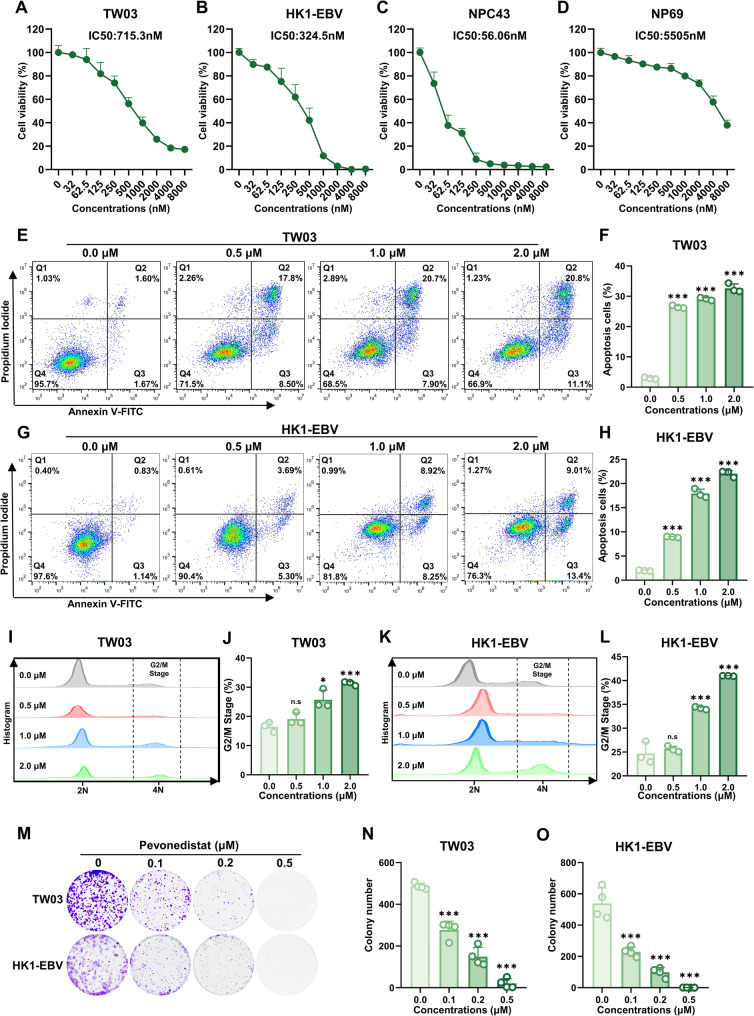
In vitro validation of the anti-nasopharyngeal carcinoma effects of Pevonedistat. **A**–**D** Cell viability of NPC cell lines (TW03, HK1-EBV, NPC43) and the nasopharyngeal epithelial cell line (NP69) following treatment with increasing concentrations of Pevonedistat, with IC50 values indicated. **E**–**H** Apoptosis of TW03 and HK1-EBV cells after 72 h treatment with increasing concentrations of Pevonedistat was assessed by Annexin V-FITC/PI staining and flow cytometric analysis (*n* = 3). **I**, **K** Representative flow cytometry histograms of cell cycle distribution in TW03 and HK1-EBV cells treated with the indicated concentrations. **J**, **L** Quantification of the percentage of cells in the G2/M phase. **M**–**O** Effects of increasing concentrations of Pevonedistat on the clonogenic capacity of TW03 and HK1-EBV cells (*n* = 4). Data are presented as mean ± SD from at least three independent experiments. Statistical analysis was performed using an unpaired two-tailed Student’s t-test. *** *P*<0.001, ** *P*<0.01, * *P*<0.05; n.s., not significant. All comparisons were made relative to the 0 µM group.

To assess the potential of Pevonedistat as a therapeutic candidate against NPC, CCK-8 assays were conducted in three NPC cell lines (TW03, HK1-EBV, NPC43) and the nasopharyngeal epithelial cell line NP69, followed by determination of their IC50 values. As illustrated in Fig. 5A–D, Pevonedistat potently inhibited cell viability, with IC50 values of 715.3 nM in TW03, 324.5 nM in HK1-EBV, 56.06 nM in NPC43, and 5,505 nM in NP69 cells. These results demonstrate that Pevonedistat exhibited substantially lower IC50 values in NPC cell lines (TW03, HK1-EBV, and NPC43) compared with the NP69 cell line, supporting its high efficacy and low toxicity profile. Apoptosis was then quantified by flow cytometry in TW03 and HK1-EBV cells after 72 h of Pevonedistat exposure. As shown in Fig. 5E–H, the apoptosis rate of both NPC cell lines increased progressively with Pevonedistat concentrations ranging from 0.5 µM to 2.0 µM. These findings indicate that Pevonedistat induces apoptosis in nasopharyngeal carcinoma cells in a dose-dependent manner. Similarly, cell cycle distribution in TW03 and HK1-EBV cells was examined after 48 h of Pevonedistat treatment. As shown in Fig. 5I–L, Pevonedistat increased the proportion of TW03 and HK1-EBV cells in the G2/M stage (4 N peak), indicating impaired cell division and consequent cell cycle arrest (The original images are presented in Supplementary Fig. 8). In addition, the morphology of TW03 cells after 24 h and 48 h of Pevonedistat treatment is shown in Supplementary Fig. 9, where cell enlargement due to cell cycle arrest and an increased number of floating cells caused by apoptosis were observed. Finally, the impact of varying concentrations of Pevonedistat on the colony-forming ability of TW03 and HK1-EBV cells was assessed. As illustrated in Fig. 5M–O, colony formation by TW03 and HK1-EBV cells declined progressively as the concentration of Pevonedistat increased, and was almost eliminated at 0.5 µM, demonstrating that Pevonedistat potently inhibits TW03 and HK1-EBV cell growth and exerts a notable anti-nasopharyngeal carcinoma effect. In summary, we evaluated the pharmacological effects of Pevonedistat on nasopharyngeal carcinoma cell lines in vitro in this section. Collectively, our findings indicate that Pevonedistat promotes apoptosis, triggers cell cycle arrest, and suppresses colony formation in nasopharyngeal carcinoma cells, supporting its strong pharmacological activity against NPC.

### Preclinical evaluation of Pevonedistat as a therapeutic agent for NPC

**Fig. 6:**
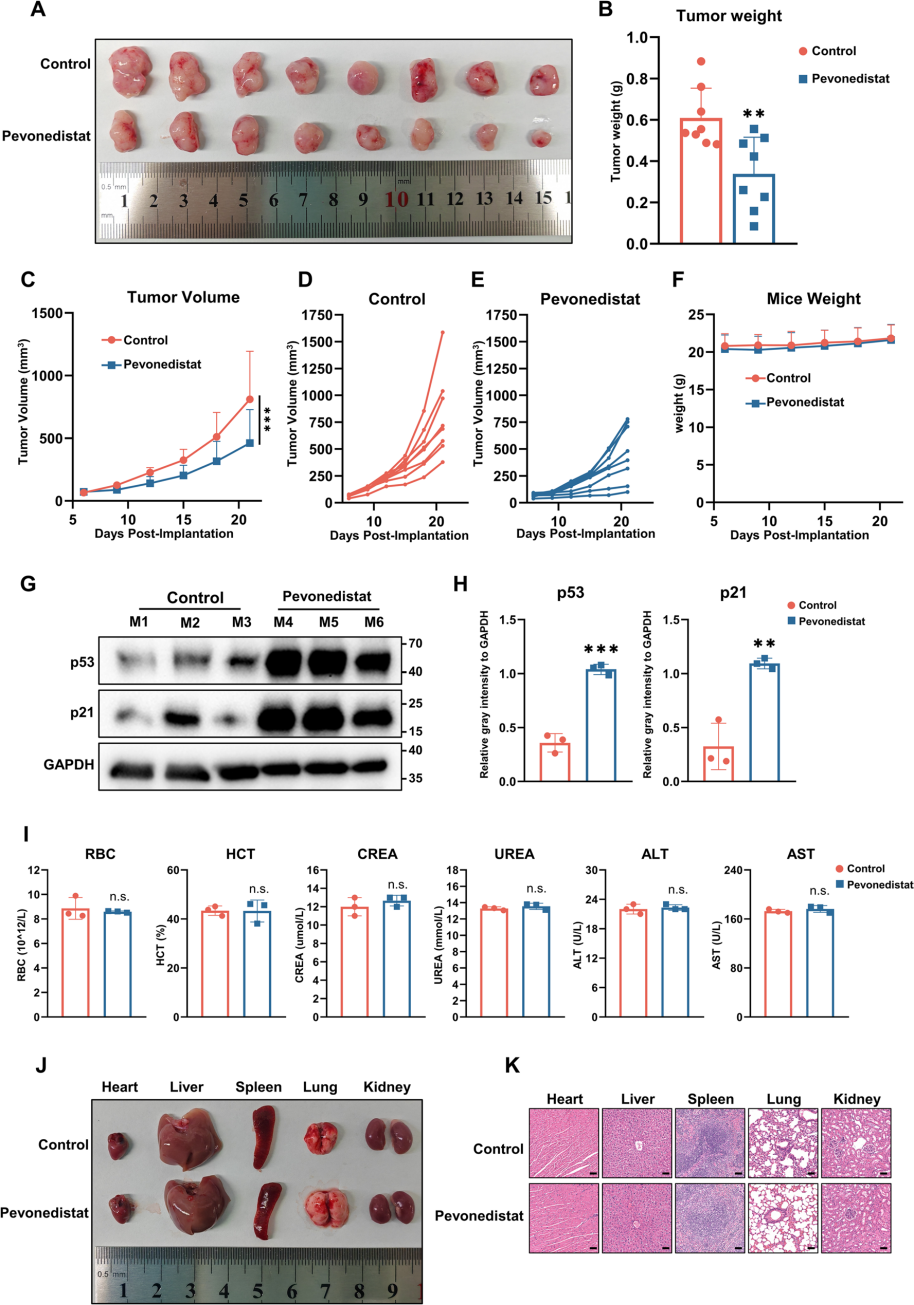
Preclinical evaluation of Pevonedistat as a therapeutic agent for nasopharyngeal carcinoma.** A** Representative tumor morphology of the control and Pevonedistat groups (*n* = 8). **B** Statistical analysis of tumor size in the control and Pevonedistat groups (*n* = 8; ** P<0.001). **C** Tumor growth curves of the control and Pevonedistat groups (*n* = 8; *** P<0.001). **D**, **E** Individual tumor growth profiles of eight tumor-bearing mice in the control and Pevonedistat groups. **F** Body weight monitoring of mice in the control and Pevonedistat groups during the experiment (*n* = 8). **G** Representative Western blot analysis of p53 and p21 expression in tumor tissues derived from xenograft models. Tumor samples were collected from three representative mice in the control group (M1–M3) and three in the Pevonedistat-treated group (M4–M6). GAPDH was used as the loading control. **H** Quantification of p53 and p21 protein expression levels. Gray intensities were quantified by densitometric analysis and normalized to GAPDH. Data are presented as mean ± SD. *** *P*<0.001, ** *P*<0.01, compared with the control group. **I** Hematological and serum biochemical analyses, including red blood cell count (RBC), hematocrit (HCT), creatinine (CREA), blood urea nitrogen (UREA), alanine aminotransferase (ALT), and aspartate aminotransferase (AST), n.s., not significant. **J** Gross morphology of major organs (heart, liver, spleen, lung, and kidney) from control and Pevonedistat-treated mice. **K** Histopathological sections of major organs (heart, liver, spleen, lung, and kidney) from control and Pevonedistat-treated mice

We evaluated the antitumor efficacy and safety of Pevonedistat in a patient-derived xenograft model of nasopharyngeal carcinoma. As mentioned above, treatment was initiated on day 6 after C17-PDX implantation, administered once every three days, with tumor volumes measured throughout the study and the experiment terminated on day 21. Tumors in the Pevonedistat-treated group were visibly smaller than those in the control group (Fig. 6A), and tumor weight analysis further revealed a significant reduction in the treatment group compared with controls (Fig. 6B). The tumor growth curves during the entire study period are shown in Fig. 6C, and Fig. 6D–E illustrate the individual tumor growth profiles of eight mice in each group. Collectively, these results demonstrate that Pevonedistat markedly suppressed the growth of C17-PDX tumors, supporting its potent in vivo antitumor activity against nasopharyngeal carcinoma. To further confirm the involvement of the CRL4-DTL-p21/p53 axis in vivo, Western blot analysis was performed on tumor tissues obtained from xenograft models. Compared with the control group, tumors treated with Pevonedistat exhibited significantly increased expression levels of p21 and p53. These results were consistent with the observations at the cellular level, further supporting that Pevonedistat exerts its anti-tumor effects, at least in part, through modulation of the CRL4-DTL-p21/p53 axis in vivo (Fig. 6G–H). We subsequently evaluated the potential adverse effects of Pevonedistat in the C17-PDX model. As shown in Fig. 6F, body weight remained stable and comparable between the control and treatment groups throughout the study. Hematological and serum biochemical parameters, including red blood cell count (RBC), hematocrit (HCT), creatinine (CREA), blood urea nitrogen (UREA), alanine aminotransferase (ALT), and aspartate aminotransferase (AST), are presented in Fig. 6G. No significant differences were observed between groups, indicating that Pevonedistat did not exert noticeable effects on hematological or biochemical indices at the tested dose. Furthermore, Fig. 6H and I show the gross morphology and histopathological sections of major organs (heart, liver, spleen, lung, and kidney), which revealed no apparent abnormalities, suggesting minimal organ toxicity. Collectively, these findings demonstrate that Pevonedistat confers potent in vivo antitumor efficacy while exhibiting limited hematological and organ toxicity, underscoring its promise as a therapeutic candidate for nasopharyngeal carcinoma.

## Discussion

Given the current limitations and adverse effects of NPC treatments, the search for safe and effective therapeutic targets and agents is urgently needed [37]. In this study, we systematically identified and evaluated novel therapeutic targets and drugs for NPC by integrating multi-level bioinformatics analyses, machine learning–based screening, functional validation, and preclinical assessment. Our study yielded several key observations. First, integrated bioinformatics analyses of multiple transcriptomic datasets identified a set of genes closely associated with NPC. Second, machine learning methods, including LASSO regression, random forest, and SVM-RFE, identified *ECT2*,* ANLN*,* DTL*, and *UBE2T* as hub genes with high diagnostic and prognostic value. Third, experimental knockdown of *DTL* in NPC cells resulted in pronounced cell cycle arrest and induction of apoptosis, confirming its functional importance. Fourth, mechanistic investigations revealed that Pevonedistat, a NEDD8-activating enzyme inhibitor, impaired DTL function and stabilized its downstream substrates p21 and p53 [38, 39]. Finally, both in vitro assays and PDX models demonstrated significant antitumor activity of Pevonedistat, supporting its preclinical therapeutic potential in NPC. In summary, these results delineate a coherent pathway from target discovery to pharmacologic validation. To our knowledge, this is the first comprehensive study linking DTL dysregulation to NPC and exploring Pevonedistat as a potential therapeutic agent for this malignancy.

DTL is the substrate receptor of the Cullin-RING E3 ubiquitin ligase 4 (CRL4) complex [40, 41]. By associating with the CRL4 complex, DTL mediates ubiquitin ligase activity [42]. Previous studies have reported that DTL is highly expressed in multiple cancers, including breast, gastric, and liver cancers [41, 43, 44]. DTL enhances cancer cell proliferation and modulates DNA damage responses by promoting p21 degradation, thereby conferring genomic instability to tumor cells and rendering it a potential oncogene [45–47]. DTL plays a crucial role in cell cycle regulation, and its dysregulation often results in cell cycle disruption. Specifically, DTL regulates DNA replication and cell cycle progression by mediating the degradation of CDT1 and p21, a cyclin-dependent kinase inhibitor [48–50]. It is well established that cell cycle dysregulation plays a pivotal role in the initiation and progression of NPC [51]. Previous studies have shown that cell cycle regulators such as *CCND1*,* CDK4/6*,* ATR*, and *CHEK1* are critical in promoting uncontrolled proliferation and malignant progression in NPC [52–54]. Nevertheless, despite well-documented evidence supporting the function of DTL as a component of the CRL4–DTL complex in cell cycle regulation, its significance in NPC has received little attention. Therefore, our identification of DTL as a hub gene in NPC fills this critical gap. Notably, although DTL overexpression and its oncogenic mechanisms have been reported in breast, gastric, and hepatocellular carcinomas, its specific contribution to NPC pathogenesis has not been clarified.

Pevonedistat, initially identified as a NEDD8 inhibitor, suppresses NEDD8 activity through covalent adduct formation, thereby leading to the inactivation of downstream CRL4 complexes [55, 56]. Our molecular docking analysis revealed that Pevonedistat exhibited the most favorable binding affinity with NEDD8 (–7.3 kcal/mol), consistent with previously reported findings. Moreover, docking predictions indicated that Pevonedistat forms stable hydrogen bonds with NEDD8 residues Gln40, Arg42, Ala72, and Arg74, suggesting that these amino acids may be critical for covalent adduct formation and functional inhibition of NEDD8.

DTL exerts its oncogenic function primarily by facilitating the ubiquitin-mediated degradation of essential cell cycle regulators [57]. Within the CRL4–DTL complex, DTL directs the proteasomal degradation of substrates including CDT1, p21, and p53 regulatory proteins [58]. Overexpression of DTL in NPC cells likely results in rapid degradation of p21 and destabilization of the p53 pathway, thereby promoting unchecked cell cycle progression and escape from apoptosis. This hypothesis was supported by our functional assays (Figs. 3G–R and 4B), which demonstrated that DTL silencing reinstated p21 and p53 stability and induced cell cycle arrest and apoptosis. By inhibiting NEDD8 activation, Pevonedistat disrupts CRL4–DTL complex activity, thereby impairing the ubiquitination and degradation of p21 and p53 and phenocopying, at least in part, the effects of DTL silencing [59]. Consistently, Pevonedistat treatment resulted in stabilization of p21 and p53 protein levels and was accompanied by cell cycle arrest and apoptosis in NPC cells (Figs. 4F and 5E–L). Together, these findings support a working model in which the antitumor effects of Pevonedistat in NPC are mediated, at least in part, through functional inhibition of the CRL4–DTL–p21/p53 axis.

Distinct from studies confined to biomarker identification, our work establishes a bridge between target discovery and therapeutic application and demonstrates the anti-NPC efficacy of Pevonedistat in PDX models, thereby highlighting its translational potential. Currently, the management of NPC primarily depends on radiotherapy, chemotherapy, and immunotherapy. While these modalities achieve favorable outcomes in early-stage disease, their efficacy is frequently limited in advanced or recurrent cases [60, 61]. Moreover, radiochemotherapy is often associated with severe adverse effects, leading to a marked reduction in patients’quality of life [62]. Our findings demonstrate that inhibition of DTL restores cell cycle checkpoints and apoptotic pathways, thereby enhancing tumor susceptibility to DNA damage-based therapies and providing a rationale for combination with radiochemotherapy. Recent studies have suggested that nasopharyngeal carcinoma is increasingly recognized as a complex and adaptive disease shaped by tumor–virus–immune interactions and therapeutic selection pressures [63]. From this ecological perspective, our findings identify the CRL4–DTL–p21/p53 axis as a targetable molecular node that constrains the proliferative fitness of NPC cells within a broader tumor ecosystem. Notably, Pevonedistat has already undergone clinical trials in other malignancies, including lung cancer and leukemia [64–67]. Its potential application in NPC could therefore expedite clinical translation.

Although this integrative strategy provides important advantages, several limitations remain. First, while multiple independent datasets were incorporated into our bioinformatics analysis, the clinical significance of DTL expression requires validation in large-scale patient cohorts. Second, the in vitro and in vivo models employed were restricted to cell lines and xenografts, which may not adequately reflect the heterogeneity of clinical tumors. Third, although the CRL4–DTL–p21/p53 axis was the primary focus of this study, DTL also modulates additional substrates involved in DNA replication and repair, which may likewise contribute to NPC development. Lastly, while Pevonedistat showed robust antitumor effects in preclinical models, issues concerning its long-term safety, resistance mechanisms, and optimal administration in NPC remain to be clarified.

## Conclusion

In summary, through comprehensive multi-layered bioinformatics analyses and validation, we identified DTL as a critical oncogenic driver in NPC and suggested Pevonedistat as a promising therapeutic candidate against this pathway. Combining bioinformatics, machine learning, molecular and cellular experiments, and preclinical assessments, we provide a holistic framework to support target identification and drug development in NPC. Collectively, our findings enhance the understanding of NPC pathogenesis and offer a tangible pathway toward novel therapeutic strategies that may eventually improve patient outcomes.

## Supplementary Information

Supplementary Material 1.

Supplementary Material 2.

## Data Availability

The datasets used and/or analyzed during the current study are available from the corresponding author on reasonable request. The key raw data of this work have been deposited in the Research Data Deposit public platform (www.researchdata.org.cn, accession number: RDDB2025245515).

## Abbreviations

ALT: alanine aminotransferase
AST: aspartate aminotransferase
AUC: area under the curve
CCK: Cell Counting Kit–8
CREA: creatinine
DEGs: differentially expressed genes
EBV: Epstein–Barr virus
FBS: fetal bovine serum
GO: Gene Ontology
HCT: hematocrit
KEGG: Kyoto Encyclopedia of Genes and Genomes
MCC: maximal clique centrality
MNC: maximum neighborhood component
NPE: nasopharyngeal epithelium
NPC: nasopharyngeal carcinoma
PDX: patient–derived xenograft
PI: propidium iodide
PPI: protein–protein interaction
RBC: red blood cell count
ROC: receiver operating characteristic
scRNA: seq–single–cell RNA sequencing
SVM: RFE–support vector machine–recursive feature elimination
UREA: blood urea nitrogen
WGCNA: weighted gene co–expression network analysis
