# Supplementary material for: Integrated bioinformatics analysis and experimental validation reveal Pevonedistat as a promising therapeutic agent modulating the CRL4–DTL–p21/p53 axis in nasopharyngeal carcinoma
Source: Hereditas. 2026 Mar 7;163:50. doi: 10.1186/s41065-026-00661-2 (PMC13081564; doi:10.1186/s41065-026-00661-2)
Supplement: Supplementary file 1 — Supplementary Material 1. [file 41065_2026_661_MOESM1_ESM.pdf]

Supplementary Figure 1

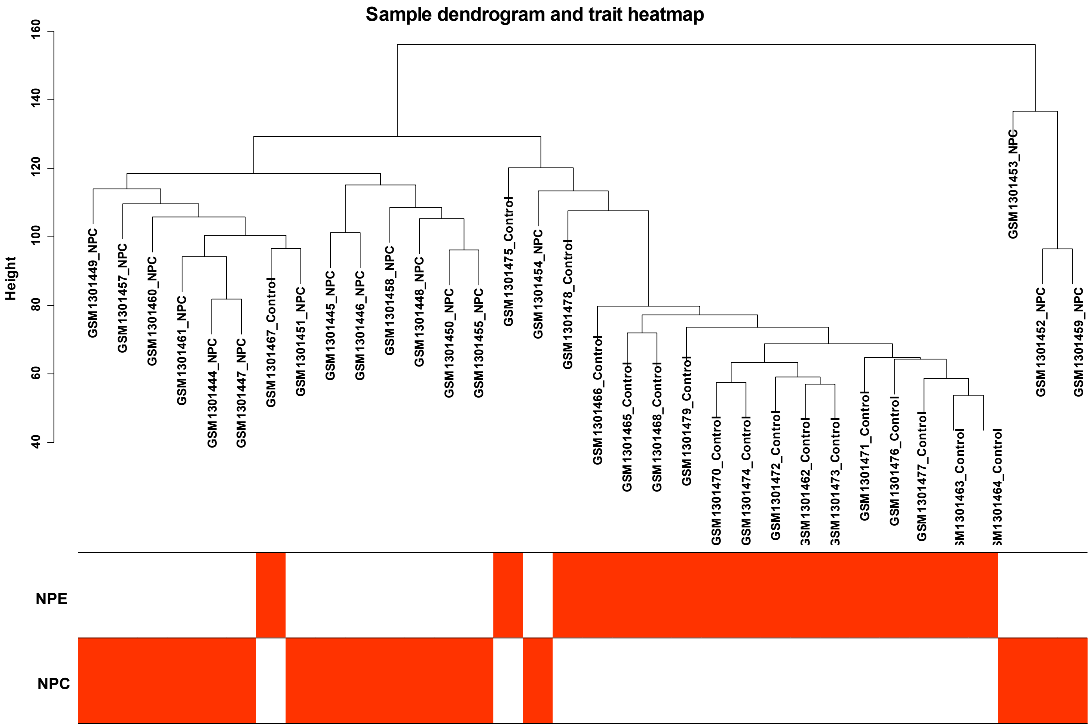

Supplementary Figure 1. Hierarchical clustering of NPC and NPE samples based on gene expression profiles. The heatmap indicates trait assignment (red = presence, white = absence), showing clear separation between NPC and NPE groups without obvious outliers.

Supplementary Figure 2

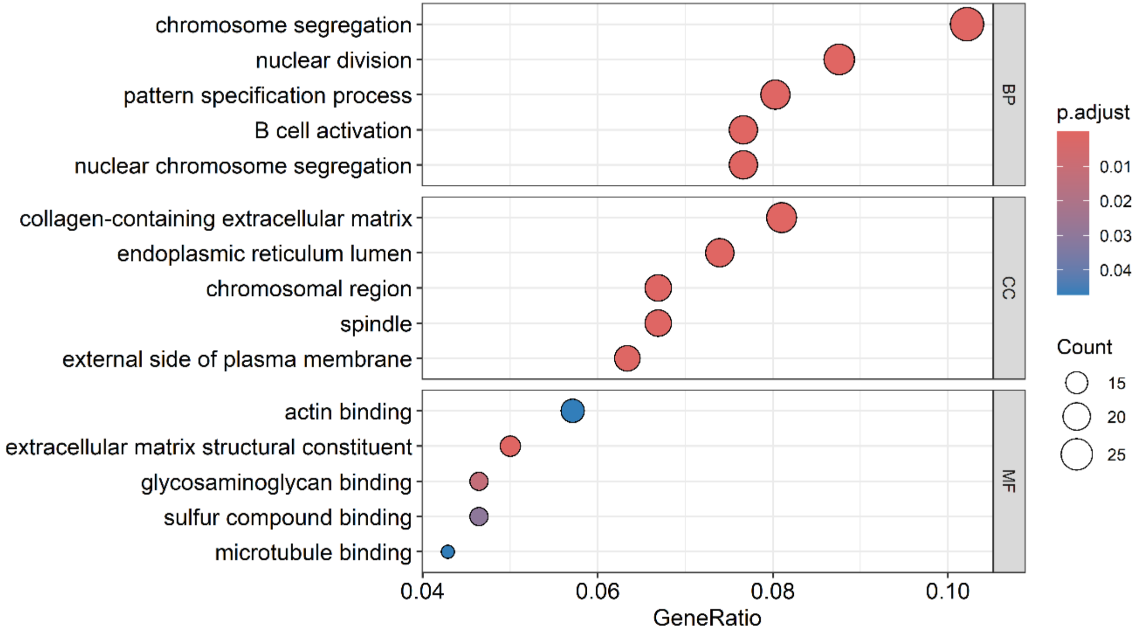

Supplementary Figure 2. Bubble plot showing the top significantly enriched GO terms in biological process (BP), cellular component (CC), and molecular function (MF) categories. The x-axis represents the GeneRatio, bubble size corresponds to the number of genes, and bubble color indicates the adjusted *P* value.

Supplementary Figure 3

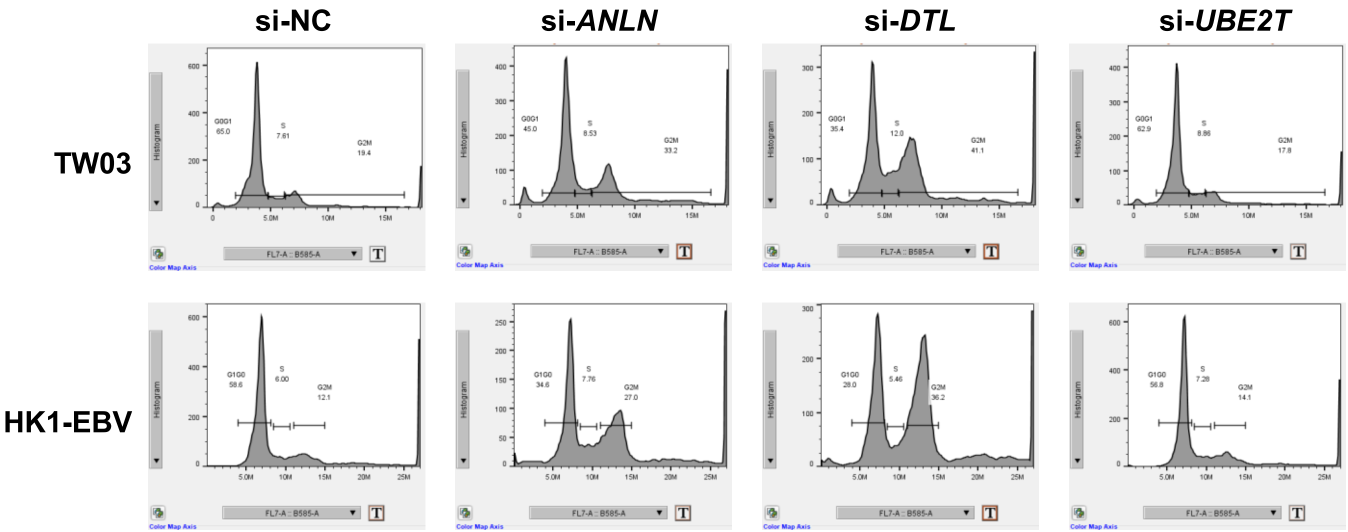

Supplementary Figure 3. Representative DNA content histograms of nasopharyngeal carcinoma cells following gene knockdown.TW03 and HK1-EBV cells were transfected with control siRNA (si-NC) or siRNAs targeting ANLN, DTL, or UBE2T, followed by flow cytometry–based cell cycle analysis using propidium iodide staining. Representative DNA content histograms are shown, illustrating the distribution of cells in G0/G1, S, and G2/M phases for each knockdown condition. These histograms represent the raw data used for quantitative cell cycle analyses presented in the main figures.

Supplementary Figure 4

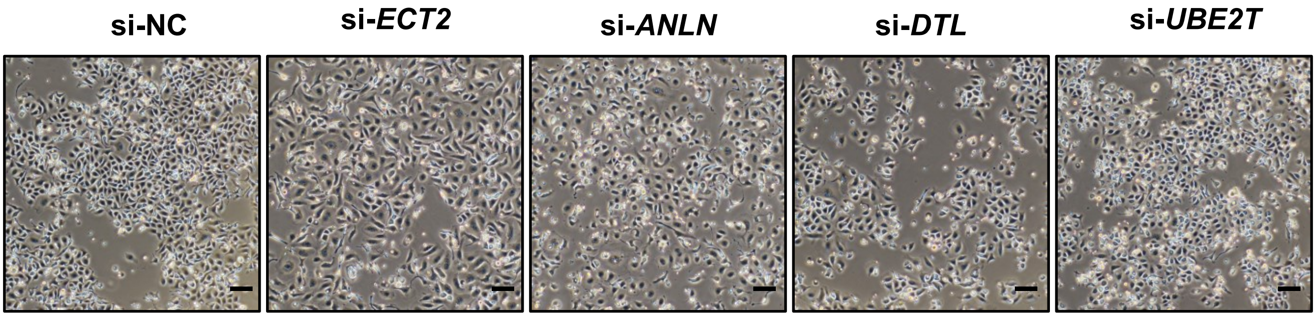

Supplementary Figure 4. Representative images of cell morphology at 48 h after siRNA-mediated knockdown of the four indicated genes (*ECT2*, *ANLN*, *DTL*, and *UBE2T*). Among them, the *DTL* knockdown group exhibited the most impaired cellular morphology with markedly reduced density. Scale bar = 200 µm.

Supplementary Figure 5

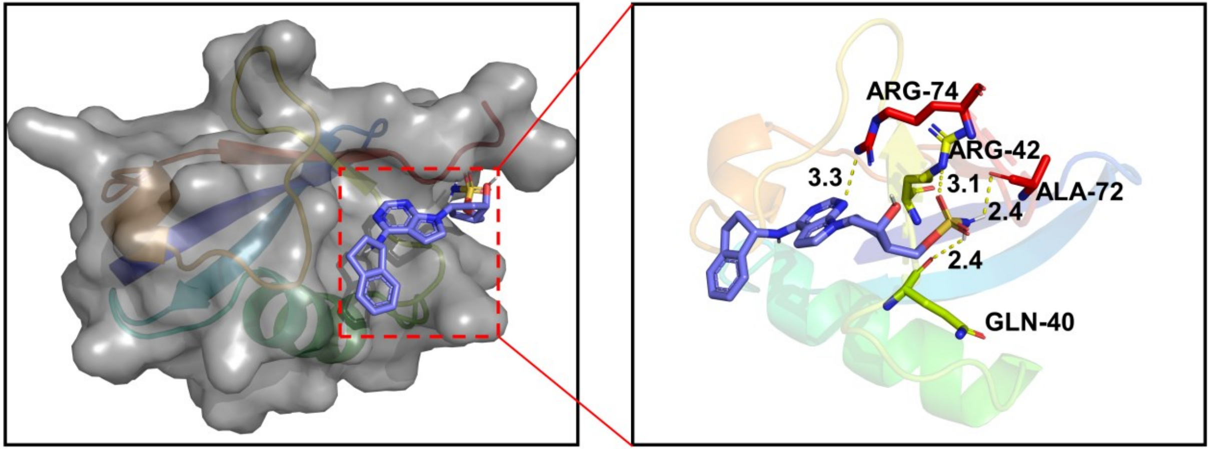

Supplementary Figure 5. Structural representation of the Pevonedistat–NEDD8 binding mode, highlighting critical residues (ARG-42, ARG-74, ALA-72, GLN-40).

Supplementary Figure 6

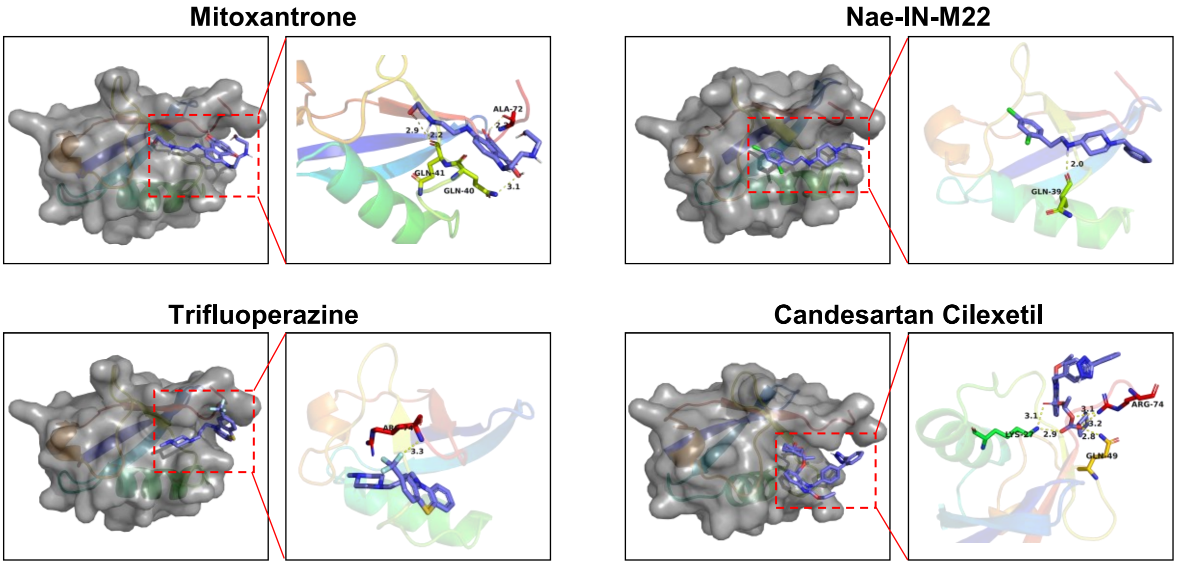

Supplementary Figure 6. Molecular docking analysis of Mitoxantrone, Nae-IN-M22, Trifluoperazine, and Candesartan Cilexetil with NEDD8, showing the interactions between the compounds and key amino acid residues of NEDD8.

Supplementary Figure 7

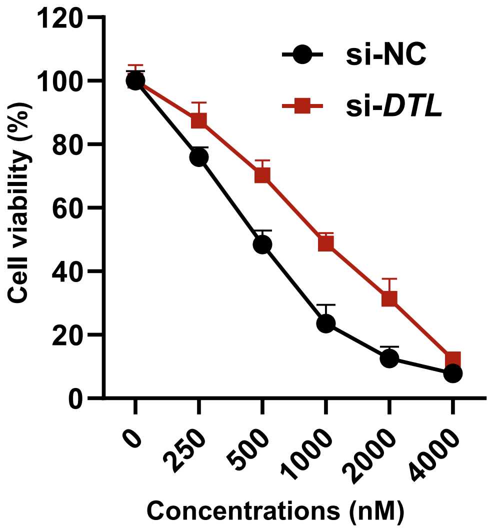

Supplementary Figure 7. Silencing DTL attenuated the cytotoxic effect of Pevonedistat on TW03 cells.

Supplementary Figure 8

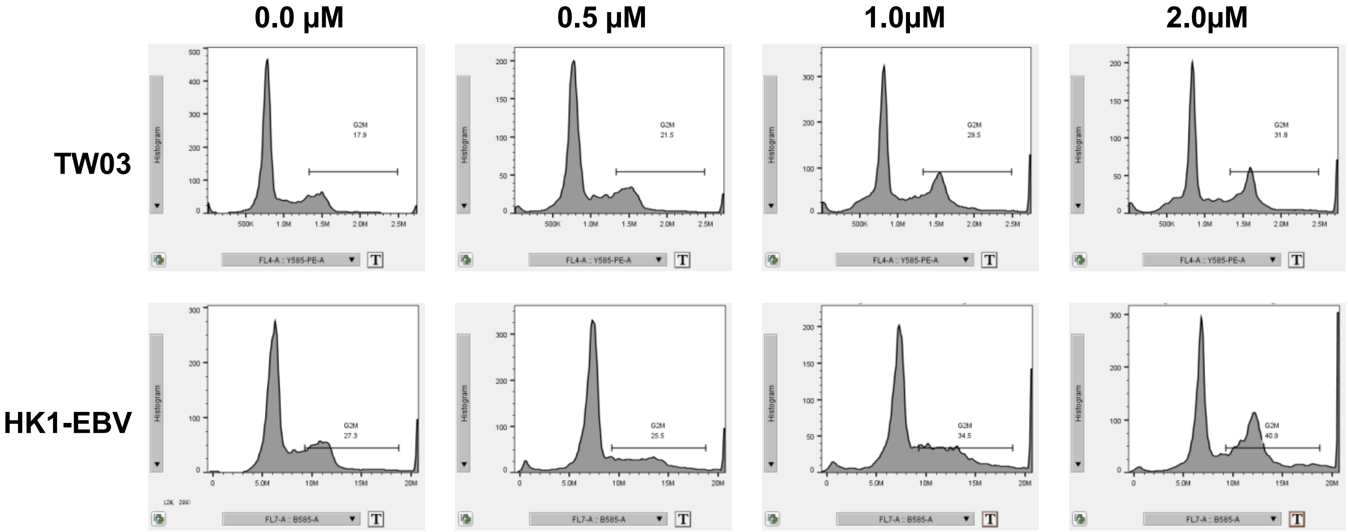

Supplementary Figure 8. Representative DNA content histograms for cell cycle analysis following Pevonedistat treatment.TW03 and HK1-EBV cells were treated with increasing concentrations of Pevonedistat (0, 0.5, 1.0, and 2.0 µM) and subjected to flow cytometry–based cell cycle analysis using propidium iodide staining. Representative DNA content histograms are shown, illustrating the distribution of cells in G2/M phases at each concentration. These histograms represent the raw data used for quantitative cell cycle analysis presented in the main figures.

Supplementary Figure 9

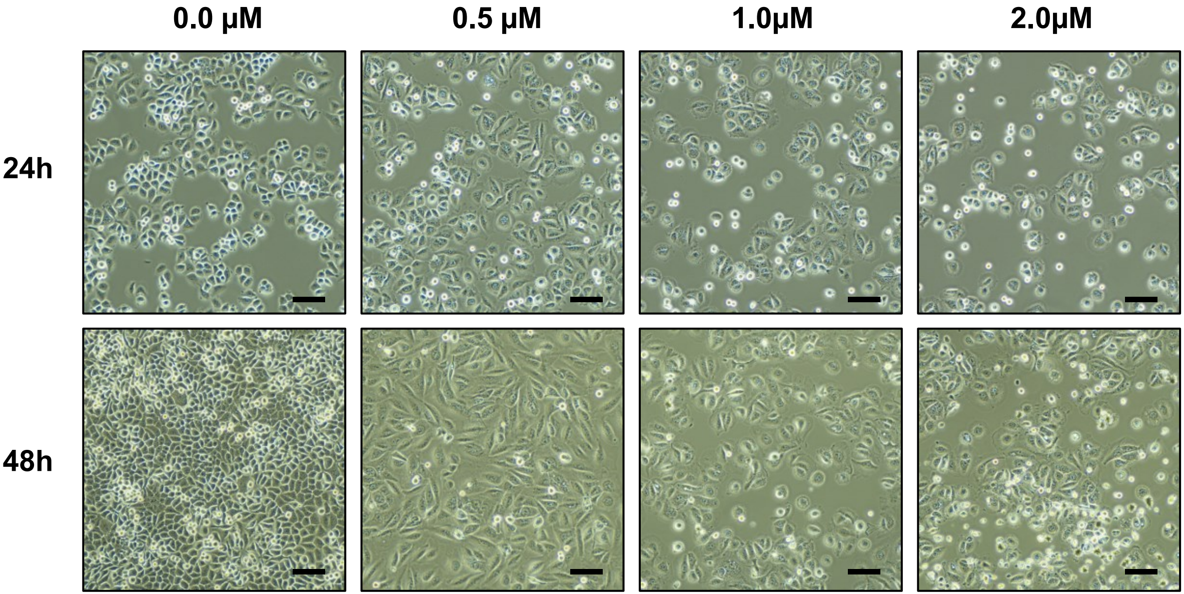

Supplementary Figure 9. Representative images of TW03 cells after treatment with graded concentrations of Pevonedistat for 24 h and 48 h. Scale bar = 100 μm.

Supplementary Table 1

Primer Sequences：

Gene Name	Forward Sequence (5'-3')	Reverse Sequence (5'-3')
ECT2	GAGAGGAGTCGGCGTTTGAA	GTCTGCCAAGCTAGTCCTCC
ANLN	AGTGTCCACACCTAGACTGG	GATTCAGCTCGAGGGACACG
DTL	CAGCTTGGCGTCCTGAGAAA	TCCAAAAGGAGGAACTGGGAC
UBE2T	ATGTTAGCCACAGAGCCACC	ACCTAATATTTGAGCTCGCAGGT
GAPDH	CTCCTCCTGTTCGACAGTCAGC	CCCAATACGACCAAATCCGTT

Supplementary Table 2

Antibody information：

Protein name	Catalog number	Company	Dilution
p53	60283-2-Ig	Proteintech	1:2000
p21	A19094	Abclonal	1:2000
GAPDH	A19056	Abclonal	1:10000
DTL/CDT2	HA721871	HUABIO	1:2000

Supplementary Table 3

Sequences of siRNAs:

Gene Name	Sequence (5'to 3')
hsa-ECT2（ID:1894）siRNA-281	/rG//rA//rU//rU//rG//rA//rA//rA//rC//rA//rA//rG//rA//rG//rU//rG//rA//rU//rA/TT
	/rU//rA//rU//rC//rA//rC//rU//rC//rU//rU//rG//rU//rU//rU//rC//rA//rA//rU//rC/TT
hsa-ANLN（ID:54443）siRNA-212	/rG//rA//rG//rA//rG//rA//rA//rU//rC//rU//rU//rC//rA//rG//rA//rG//rA//rA//rA/TT
	/rU//rU//rU//rC//rU//rC//rU//rG//rA//rA//rG//rA//rU//rU//rC//rU//rC//rU//rC/TT
hsa-DTL（ID:51514）siRNA-2042	/rG//rC//rA//rC//rA//rU//rA//rC//rU//rU//rC//rC//rA//rU//rA//rG//rA//rA//rA/TT
	/rU//rU//rU//rC//rU//rA//rU//rG//rG//rA//rA//rG//rU//rA//rU//rG//rU//rG//rC/TT
hsa-UBE2T（ID:29089）siRNA-232	/rC//rA//rA//rC//rA//rC//rA//rC//rC//rU//rU//rA//rU//rG//rA//rG//rA//rA//rA/TT
	/rU//rU//rU//rC//rU//rC//rA//rU//rA//rA//rG//rG//rU//rG//rU//rG//rU//rU//rG/TT
