# Supplementary material for: Integrated bioinformatics analysis and experimental validation reveal Pevonedistat as a promising therapeutic agent modulating the CRL4–DTL–p21/p53 axis in nasopharyngeal carcinoma
Source: Hereditas. 2026 Mar 7;163:50. doi: 10.1186/s41065-026-00661-2 (PMC13081564; doi:10.1186/s41065-026-00661-2)

Uncropped Gels and Blots images

Figure 4A.

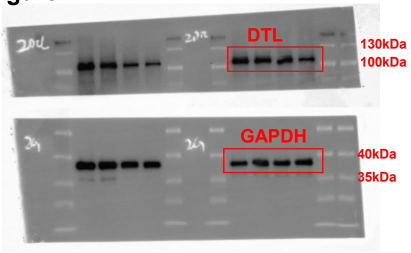

Figure 4B.

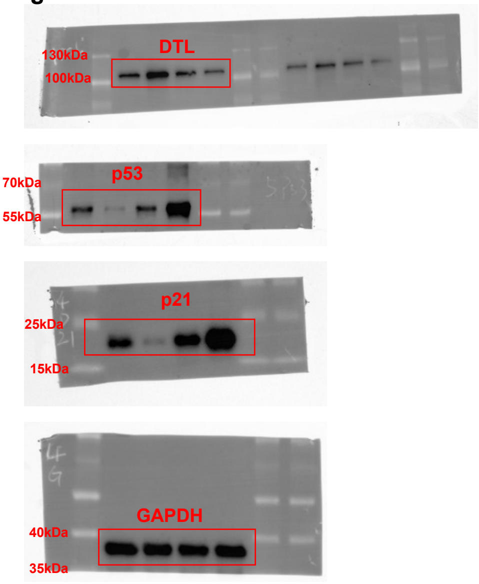

Uncropped Gels and Blots images

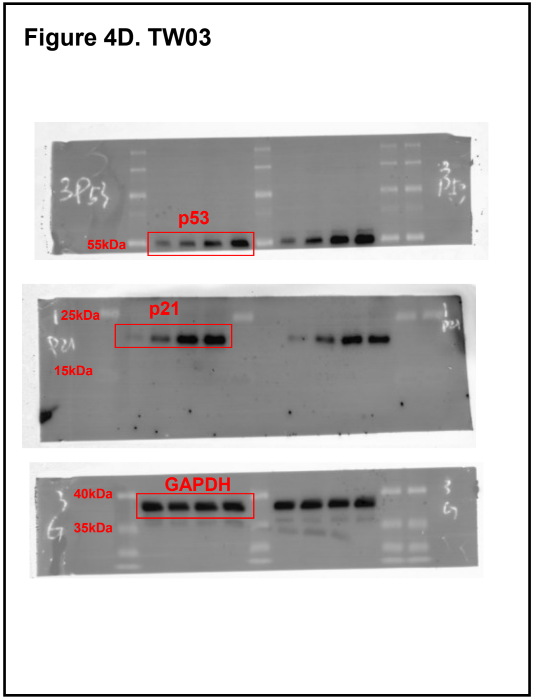

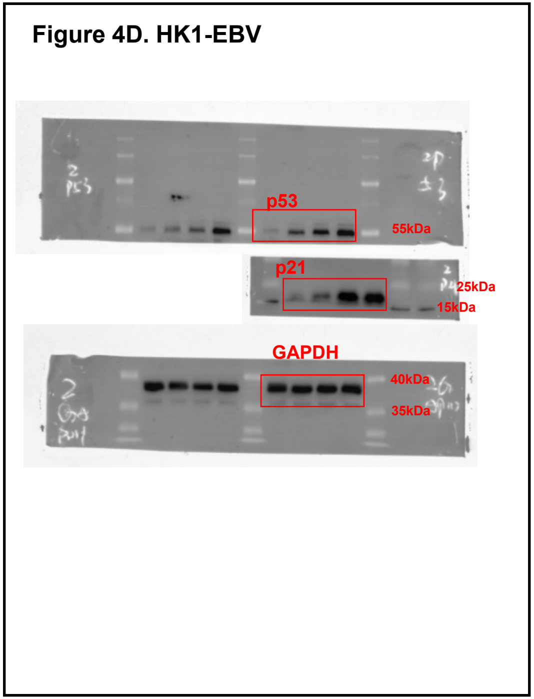

Uncropped Gels and Blots images

Figure 4E.

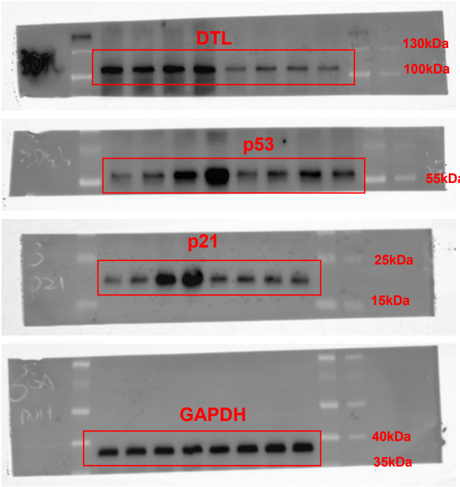

Figure 6G.

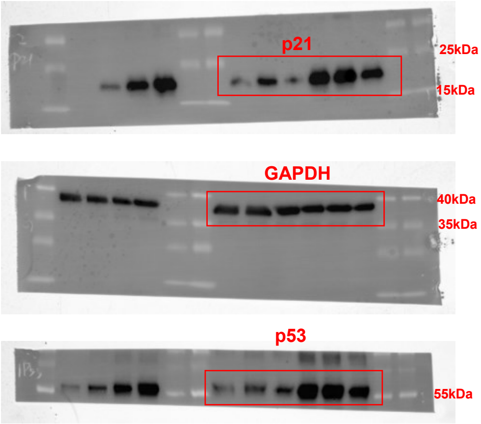

Supplement: Supplementary file 2 — Supplementary Material 2. [file 41065_2026_661_MOESM2_ESM.pdf]
